# A systematic review evaluating imaging techniques to determine chronicity of deep vein thrombosis

**DOI:** 10.1177/02683555251358915

**Published:** 2025-07-16

**Authors:** Kirtan D Patel, Manal Ahmad, Matthew Tan, Sarah Onida, Alun Davies

**Affiliations:** 1 Department of Surgery and Cancer, 4615 Imperial College London, London, UK; 2 Department of Vascular Surgery, Imperial College Healthcare NHS Trust, London, UK

**Keywords:** Deep vein thrombosis, duplex ultrasound, CT, MRI, VTE management

## Abstract

**Objective:**

The objective of this systematic review is to evaluate the different imaging techniques used to assess thrombus chronicity in patients with deep venous thrombosis (DVT).

**Methods:**

A systematic search of Medline, EMBASE, CENTRAL and Web of Science databases was performed to identify meta-analyses, systematic reviews, randomised controlled trials or observational studies investigating imaging techniques for assessing DVT chronicity.

**Results:**

Overall, 56 studies were reviewed. Various imaging modalities have been explored to assess DVT chronicity. Ultrasound parameters such as echogenicity and vein diameter proved unreliable, though elastography showed promise by quantifying thrombus stiffness. Computed Tomography (CT) studies were limited. In contrast, Magnetic Resonance Imaging (MRI) demonstrated potential for differentiating thrombus age, and nuclear imaging with targeted radiotracers, along with techniques like photoacoustic imaging and Optical coherence tomography (OCT), offered high diagnostic accuracy.

**Conclusion:**

This review evaluated various imaging techniques for thrombus aging. No single modality is ideal, but MRI shows the greatest potential for advancement. In select populations, it may enhance cost-effectiveness and improve outcomes for catheter-based DVT interventions.

## Introduction

Deep vein thrombosis (DVT) affects approximately 1 in 2000 patients a year, commonly presenting with pain, swelling and erythema of affected limbs.^1^ Pulmonary embolism (PE), post-thrombotic syndrome (PTS) and recurrence are well known sequelae of DVT affecting a significant proportion of DVT patients. PE represents a life-threatening complication resulting in 0.4% of deaths in the UK in 2023. Whilst PTS is not life threatening, it is a chronic condition characterised by pain, swelling, itching and ulceration with severe impact on patient quality of life and the significant costs estimated at $3000 per patient per annum.^2–6^ A quarter to half of all patients with DVT will go on to develop PTS.^7,8^ As a result, DVT is significant cause of morbidity and mortality worldwide.

The diagnosis of a DVT involves clinical assessment, use of clinical risk stratifying tools (such as the Wells score), D-dimer testing and Duplex ultrasonography.^9^ Whilst this strategy may identify a DVT, it does not provide information on the age or chronicity of a thrombus.

The need to determine the thrombus chronicity has been highlighted by the increased use of interventional techniques to reduce thrombus burden in iliofemoral DVTs. Several randomised controlled trials (RCTs) have investigated the use of catheter directed thrombolysis (CDT), mechanical or pharmaco-mechanical methods to restore flow in the iliofemoral segment.^10,11^ There is evidence to suggest that CDT may reduce the incidence and severity of PTS, with studies reporting higher recanalization rates with CDT in acute DVT (<15 days from onset of symptoms) compared to subacute (15–30 days) or DVTs older than 30 days.^12^

The efficacy of CDT is influenced by the progression of thrombus development and its cellular composition. Acute DVT is predominantly comprised of intact red blood cells and platelets entrapped within a fibrin-rich mesh. Over time, the thrombus undergoes cellular infiltration initially by neutrophils followed by monocytes, macrophages and fibroblasts, leading to replacement of fibrin with collagen. Consequently, chronic thrombi transform into fibrotic scar tissue with veins, rendering them biologically resistant to thrombolysis.^13^

Guidelines in the UK and Europe have therefore recommended that CDT only be undertaken in patients whose symptoms have been present for less than 14 days.^14,15^ However, challenges arise due to the often-non-specific nature of symptoms and the potential of DVT to develop before symptoms become apparent. Symptom duration has been shown to be an unreliable predictor of thrombus characteristics.^16^ As a result, various imaging modalities have been investigated to more accurately determine the chronicity of a thrombus.

This systematic review therefore aims to evaluate imaging techniques used for thrombus chronicity assessment and their efficacy.

## Methods

This study was conducted in adherence to the preferred Reporting Items for Systematic Reviews and Meta-Analyses (PRISMA) standards.^17^ The review was prospectively registered on PROSPERO (ID: CRD42023395900).

### Search strategy

A systematic search was conducted on 10^th^ February 2025. Databases searched included Medline (via PubMed), EMBASE (via Ovid), CENTRAL and Web of Science. No date limits were applied. Search terms included combinations of terms for DVT, imaging and staging. A full search strategy can be found in Appendix 1.

Once duplicates were excluded, two authors (KDP and MA) independently performed the title/abstract screen and subsequently the full text screen to identify relevant articles. Any disagreements were discussed to come to a consensus. Any unresolved disputes were referred to a third reviewer (SO) for resolution.

### Inclusion and exclusion criteria

RCTs and observational studies (case-control and cohort studies) investigating imaging techniques to assess DVT chronicity affecting the limbs or trunk were included. Systematic reviews and meta-analyses were also included to ensure completeness of data.

Animal studies and ex vivo studies were included to provide indirect evidence.

Studies reporting on children or adolescents (age <18 years) and those reporting on cerebral, portal or mesenteric venous thromboses were excluded. Review articles, case series and reports were also excluded.

### Data extraction

Two authors (KDP and MA) independently extracted the following data from each study: first author, year of publication, type of study, imaging modality, subtype imaging technique, comparator where applicable, number of subjects, location of DVT, measure of chronicity, sensitivity, specificity, positive predictive value, negative predictive value, area under curve, diagnostic accuracy and inter observer reliability.

## Results

### Systematic search strategy

**Figure 1.**
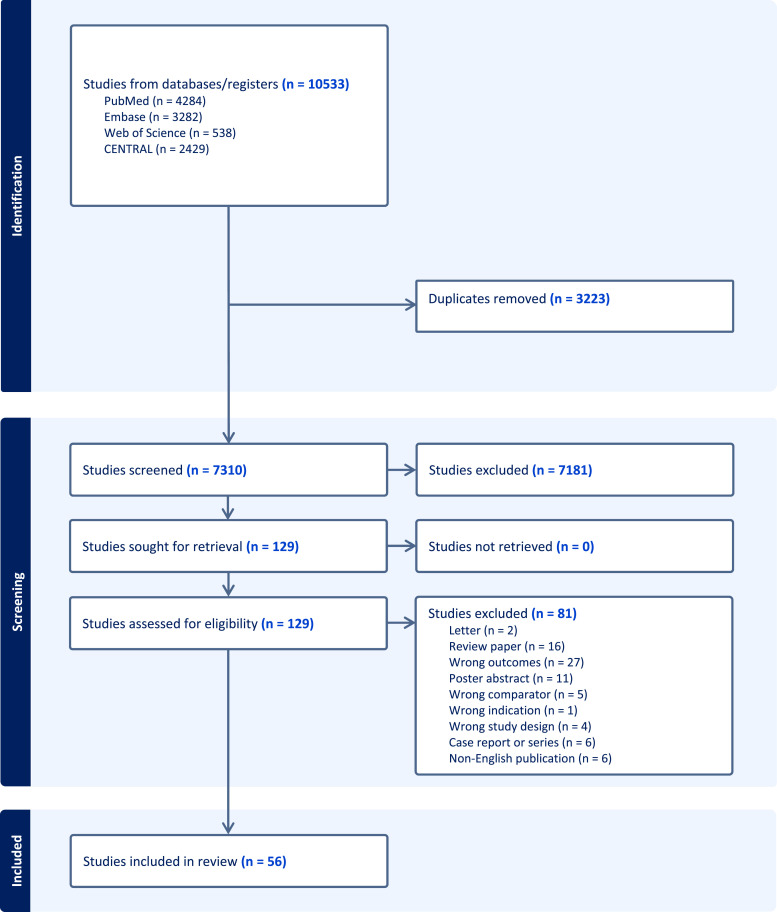
PRISMA flow diagram.

The systematic search revealed a total of 10,533 publications. A total of 3223 duplicates were removed at this stage. Following title and abstract screening, a further 7181 publications were deemed to be irrelevant and were removed. Full text review of 129 papers was undertaken of which a further 81 papers were excluded. Manual review of the references of these remaining 48 papers identified eight further publications for inclusion. In total 56 papers were included for data extraction and analysis. A PRISMA flow diagram is provided in Figure 1.

### Study demographics

Of the studies included for data extraction there were two systematic reviews, 36 human studies and 18 animal studies. Ex vivo methods were reported in five of the animal studies.

There were six broad investigation categories utilised in DVT chronicity evaluation: ultrasonography (*n* = 11), elastography (*n* = 20), computed tomography (CT) (*n* = 2), magnetic resonance imaging (MRI) (*n* = 16), nuclear medicine scans (*n* = 5) and photoacoustic imaging and optical coherence tomography (OCT) (*n* = 2).

### Ultrasound

Ultrasound was reported in 11 studies to determine DVT chronicity.

Ultrasonic features that are used to distinguish acute and more mature DVT include: echogenicity, thrombus attachment to vein wall, vein lumen size, compressibility, collateralisation, and function of the valves.^18^

#### Vein diameter

Vein diameter was observed to decrease as thrombi mature.^19,20^ When vein-to-artery ratio was calculated, acute DVT showed a significantly higher ratio compared to normal veins. While less acute DVTs tend to have a lower ratio, this difference was not statistically significant. A notable overlap of ratios was observed in acute DVT, mature DVT and normal vein groups, indicating the vein-to-artery ratios should be interpreted in conjunction with other ultrasonographic findings.^21^

#### Clot echogenicity

A single animal study investigated the use of basic ultrasonographic techniques to assess thrombus chronicity. In a porcine IVC model, thrombi at day 1, 7 and 14 days were compared, revealing that thrombus echogenicity alone was insufficient to differentiate thrombus age.^22^

In human studies, thrombus echogenicity was reported by five studies, with one study showing no correlation between thrombus echogenicity and chronicity.^19^ In the remaining four studies, echogenicity was found to have a positive correlation with thrombus chronicity.^20,23–25^ This correlation was found to be weak in two studies,^20,24^ while another reported high sensitivity (93.3%) but low specificity (57.8%).^23^ Conversely, the fourth study showed a high area under the curve (AUC) of 0.92 ± 0.04 for the receiver operating characteristic (ROC) curve for echogenicity.^25^ Overall echogenicity appears to be able identify some change in thrombus chronicity although it is neither accurate nor reliable.

#### Ultrasonic tissue characterisation

Ultrasound tissue characterisation (UTC) evaluates thrombus content by analysing attenuation and scattering of ultrasonic waves as they interact with a material. A best fit line is generated based on these measurements, where the slope represents the rate of change in ultrasound signal intensity across the thrombus. A steeper slope indicates greater heterogeneity, characteristic of less organized acute thrombi, while a flatter slope suggests homogeneity, typical of more organized chronic thrombi. The *y*-intercept reflects the initial signal intensity, with higher values indicating increased echogenicity, associated with chronic thrombi due to greater fibrin density and organization.

In a single animal study, the UTC line of best fit changed accordingly as the thrombus matured. These changes correlated with histological alterations as the fibrin mesh within the thrombus became looser and less dense. A discriminant score derived from UTC exhibited low accuracy in identifying thrombus chronicity in the early stages (46.4% on day 1) but improved significantly in later stages (87% on day 14).^22^

Similar to the findings in the animal study, UTC demonstrated a significantly lower *y*-intercept in mature DVT compared to acute DVT in humans. Additionally, utilizing the discriminant score, acute DVTs were characterized with a sensitivity of 94.7% and specificity of 90.3%.^26^

#### Grey-scale median

Grey-scale median (GSM) is an objective measure of overall echogenicity within a segmented image and has previously been used to identify high risk atherosclerotic plaques.^27,28^

It was hypothesised that as thrombus organisation progresses, both echogenicity and GSM value would increase accordingly. However, the two studies that evaluated GSM yielded mixed results.^29,30^ One study reported that acute DVT (<2 weeks) had significantly lower GSM values compared to older thrombi, with an acceptable AUC of 0.765. It was determined that, despite the ability to differentiate between acute and older thrombi, GSM exhibited poor sensitivity and specificity, resulting in poor overall accuracy.^29^

Conversely, another study found no correlation between GSM and thrombus chronicity. However, it was proposed that symptom duration may not be a reliable indicator of thrombus age as some thrombi <7 days of symptoms exhibited ultrasound characteristics associated with older, more organised thrombi.^30^

#### Ultrasound versus venography in assessing thrombus chronicity

Two studies compared ultrasound to venography, showing ultrasound to have a similar ability to detect acute and chronic changes associated with DVT.^31,32^

The CLOUT registry revealed a significant discrepancy of 17.5% in classifying thrombi as acute, subacute, or chronic when combining ultrasound, symptom duration, venography, and intravascular ultrasound, compared to visual thrombus inspection. Notably, 14.7% of DVT cases were found to be more chronic than imaging suggested. Although visual inspection of the clot is not a validated method to assess clots and may be prone to bias with variation between assessors, these findings may suggest that ultrasound imaging, regardless of modality, and venography could be unreliable in accurately determining thrombus chronicity.^16^

### Elastography

Elastography was investigated in 20 studies across in vitro, animal and human populations. Elastography is a non-invasive imaging technique that determines the mechanical properties of biological tissues. The tissues’ stiffness is determined by applying deformation forces and then strain is quantified by observing displacement through typically ultrasound of MRI. Currently, elastography is most commonly used in chronic liver disease to assess the level of fibrosis, but also has been used in breast, thyroid, kidney and prostatic evaluation.

#### Strain elastography

Two separate studies reported a murine IVC model strain analysis performed at 2, 6 and 9 days. A negative correlation between thrombus age and elasticity was demonstrated, suggesting that elastography could be used to determine thrombus chronicity, although no quantitative measures of strain were utilized in their analysis.^33,34^

Subsequent animal studies confirmed the increase in stiffness and quantified that stiffness of thrombi increased by three to six-fold between days 3 and 10, indicating a significant rise in stiffness over time.^35,36^ An exponential function was derived to predict the age of a thrombus based on the normalised strain measurement. The root mean squared error between the true age and predicted age was found to be 0.8.^37^

Strain elastography was evaluated against histological findings in a pig IVC model across two studies, both of which confirmed that normalized strain decreases with increasing thrombus age.^38,39^ A transition in thrombus stiffness was observed between days 6 and 12, corresponding to increased thrombus organization. By day six, endothelial cell proliferation and limited fibroblast infiltration were noted, whereas by day 12, extensive fibroblast and collagen infiltration, along with focal recanalization, became evident.^38,39^

There was significant heterogeneity in the reporting of strain elastography in the assessment of thrombus chronicity in humans. One study found that elastography could not distinguish between acute (<14 days) and subacute (15–28 days) thrombi.^23^ However, another study comparing acute thrombi (mean age 5.7 days) to chronic thrombi (mean age 8 months) demonstrated that acute thrombi had significantly higher normalized strain values. Normalized strain showed an AUC of 0.97 ± 0.02, with a sensitivity of 87% and specificity of 91% for identifying acute DVT.^25^ The variation in clot ages between studies complicates the interpretation and overall significance of the results. A third study utilizing the elasticity index (EI), a unitless measure of relative strain, demonstrated that acute DVT had significantly higher EI values compared to mature DVT, irrespective of thrombus location. Using ROC analysis, the sensitivity and specificity for identifying acute DVT were 98.9% and 99.1%, respectively.^40^

Strain ratios – which compare the stiffness of a clot to nearby normal tissue – increased significantly as thrombi advanced from acute to subacute and chronic stages of DVT.^41^

#### Shear wave elastography

Shear wave elastography (SWE) provides a direct quantitative measurement of tissue stiffness. Two studies evaluated SWE in thrombi involving rabbit jugular vein. Consistent with previous strain elastography findings, the studies demonstrated that stiffness increases with thrombus maturity.^42^ Elasticity measurements indicated a transition from acute DVT to subacute DVT at day 4 and to mature DVT at day 7.^42,43^ These findings were further validated through ex vivo analysis of the thrombi^43^

SWE has been further evaluated in six human studies, all of which demonstrated a positive correlation between stiffness and clot chronicity.^20,24,44–47^ The reported AUC values ranged from 0.85 to 0.976, with sensitivity varying between 82.5% and 96.3% and specificity ranging from 73.0% to 91.3%.^20,24,45^

SWE has been combined with super microvascular imaging (SMI) to enhance diagnostic accuracy. SWE demonstrated high accuracy in distinguishing acute DVT from mature DVT, while SMI combined with SWE was effective in detecting microvascular flow within subacute thrombi, indicative of internal lysis with an AUC of 0.89 and accuracy 86.86%.^24^

When thrombi were evaluated in the context of CDT, thrombi with lower SWE values were associated with greater treatment success.^46^ Additionally, thrombi treated with CDT, irrespective of their age, exhibited lower SWE values compared to those treated solely with anticoagulation.^47^

### Computed tomography (CT)

Two studies investigated the use of CT imaging to assess DVT chronicity.

One study found that 61% of venous segments showed complete resolution after thrombolysis and 35% after anticoagulation, with mature DVT indicators including truncal vein luminal obliteration, fibrotic bands, decreased venous calibre, and collateral vein formation.^48^ Another study recognised the obturator hook sign on CT venography as a marker for chronic iliofemoral outflow obstruction, with 38 out of 40 cases associated with mature DVT.^49^

### Magnetic resonance venography (MRV)

MRI was investigated in 16 studies to determine the age of thrombi.

#### Non-contrast MRV

In the rabbit internal jugular vein model, MRI results were compared with histological analysis of the clot. Over time, from 4 h to 4 weeks, signal intensity on T2-weighted images decreased, along with a reduction in T2 and T1 relaxation times of the clot.^50^

On diffusion weighted imaging (DWI), the contrast-to-noise ratio (CNR) was increased with maturity of the thrombus.^51^ These changes correlated with the more acute thrombi, which contained a higher abundance of erythrocytes, being progressively replaced by smooth muscle cells, macrophages, iron, and collagen over time.^50,51^

A more recent study found in a porcine model no significant difference in clot chronicity when using T1 relaxation times without contrast.^52^

In Behcet’s patients it was shown that non-contrast MRV was superior to Doppler Ultrasound at detecting mature DVT and its features.^53^

#### Contrast enhanced MRV (CE-MRV)

Upon contrast administration in a porcine model, T1 relaxation times were significantly higher in acute thrombi compared to subacute and chronic thrombi. Similarly, T2 relaxation times were higher in acute thrombi than in subacute thrombi, but no difference was observed between acute and chronic thrombi.^52^

Magnetization transfer (MT) detects protons bound to macromolecules. As thrombi mature and become more organized, the macromolecular content increases, leading to higher MT values. The apparent diffusion coefficient (ADC), derived from diffusion-weighted imaging (DWI), measures how freely water molecules move within tissue. Acute thrombi typically show higher ADC values due to looser structure, while more chronic, densely packed thrombi should exhibit lower ADC values.

Utilising fibrin-binding gadolinium-based contrast agents in a rat model, revealed MT and DWI could assess clot protein composition.^54,55^ MT was notably higher in more mature thrombi, showing a positive correlation with protein content, indicating its potential to differentiate collagen-rich chronic thrombi from those composed primarily of erythrocytes and fibrin.

ADC was low in early and late thrombi, peaking in intermediate aged thrombi, suggesting that erythrocytes in early thrombi and collagen in older thrombi hinder water diffusion. MT demonstrated 92% sensitivity and 100% specificity in distinguishing thrombi younger or older than 14 days, while ADC could not. Combining MT and ADC improved sensitivity and specificity for identifying intermediate thrombi. R1 relaxation rate correlated positively with fibrin content and gadolinium in the thrombus, with shorter R1 times indicating more mature thrombi. R1 relaxation rate was also predictive of successful thrombolysis, with 94% sensitivity and 99% specificity for predicting lysis success.^54^

One study used a combination of criteria, including vein diameter, venous lumen intensity, recanalization, and fibrosis, to assess clot chronicity using CE-MRV. The results showed excellent interobserver reliability (κ = 0.97) between experts and good reliability (κ = 0.82) between experts and inexperienced radiologists. The average symptom duration aligned well with the categorised clot types determined by CE-MRV.^56^

A post-hoc analysis of patients from the CAVA trial confirmed high observer agreement (κ = 0.85). Among 56 cases evaluated, 27 were classified as acute, 17 as subacute, 12 as chronic, and 3 as acute on chronic by CE-MRV.^10,57^ Notably, 91.7% of chronic cases based on CE-MRV were classified as acute or subacute by symptom duration. Thrombolysis success was higher in the acute and subacute groups compared to the chronic group (68.2% vs 16.7%) as classified by CE-MRV. The study concluded that CE-MRV is useful for determining clot characteristics for thrombolytic therapy.^57^

#### Magnetic resonance direct thrombus imaging (MRDTI)

Acute DVT exhibited hyperintense signals on T1 images using magnetic resonance direct thrombus imaging (MRDTI).^58^ These T1 hyperintensities had disappeared in 90% of patients at 3 months and all patients at 6 months though abnormalities were still visible in a third of patients on compression ultrasound at 6 months.^58^

MRDTI was shown to have similarly hyperintense signals with acute recurrent DVT whilst being normal with those with mature DVT.^59^

Consistency between non-contrast MRV and MRDTI findings appeared to be more in keeping with acute thrombi whereas greater discrepancy was associated with older thrombi.^60^

#### Cardiovascular MRI

Cardiovascular MRI (CMR) techniques were utilized to detect subacute and mature DVT, showing that CMR had similar detection rates to contrast-enhanced MRI (CE-MRV), eliminating the need for contrast and its associated risks.^61^

The relative signal intensity (rSI) of thrombus to adjacent muscle was significantly higher in acute thrombus compared to non-acute thrombus, and the ADC was significantly higher in acute thrombi. These ADC values could distinguish acute and non-acute DVT with 93% sensitivity and 90% specificity.^59,62^

Further work showed that T1 mapping was more suitable for staging DVT compared to rSI measurement, providing greater interobserver reliability, sensitivity, and specificity.^63^

#### Specific MRV features

Various features on MRV have been studied to try and discriminate acute and mature DVT. Presence of collateral veins had a 97% sensitivity and 83% specificity to diagnose mature DVT (>21 days). Whilst subcutaneous honeycombing had a sensitivity of 94% and specificity of 90%.^64^

Veins containing acute thrombus had a greater diameter than normal veins and thrombi displayed a peripheral rim of enhancement which was referred to as a “bull’s-eye sign”. Utilising this sign as a rim centre ratio, thrombi within the first 14 days of diagnosis were shown to have a significantly higher ratio compared to those older than 14 days.^65^

### Nuclear medicine

Nuclear imaging was used to determine chronicity in 5 studies. All studies were performed on human subjects. Technetium 99m (^99m^Tc) scintigraphy (4 studies) and ^18^F-FDG PET (1 study) were the techniques used in these studies.

Each of the ^99m^Tc scintigraphy studies used different radiolabelled markers.

^99m^Tc radiolabelled apcitide targets glycoprotein IIb/IIIa, which is expressed on activated platelets that are abundant in acute DVT. The sensitivity and specificity for detecting acute DVT were 92% and 82%–90%, respectively, with excellent inter-rater reliability (κ = 0.87) between two experts.^66^

The use of ^99m^Tc radiolabelled recombinant tissue plasminogen activator (rt-PA) was investigated in 55 patients with acute DVT, with scans performed at 24 h, 7 days, and 30 days. At day 7, 72% of patients with persistent thrombi showed rt-PA uptake, but no uptake was observed at day 30.^67^

^99m^Tc radiolabelled HMPAO leukocyte scintigraphy was used in 45 patients diagnosed with DVT. Elevated isotope levels were observed in limbs with active thrombosis at diagnosis and in 93% of patients at 8 weeks. The target-to-background ratio of the isotope decreased significantly from 7.43 to 4.27 n/cm^2^.^68^

^99m^Tc radiolabelled heparin along with maximum venous outflow (MVO) was assessed in 17 patients with acute DVT diagnosed via X-ray venography. Patients with acute DVT had an MVO <60 mL/min/100 mL, while mature DVT showed irregular venous filling and a higher MVO in some cases.^69^

The use of 18F-FDG PET to determine DVT chronicity was investigated in 12 patients with acute DVT and 24 controls. The study found that metabolic activity (SUVmax) was significantly higher in thrombosed segments compared to matched non-thrombosed segments in the contralateral leg and control patients. Additionally, a negative correlation was observed between metabolic activity and time from the onset of symptoms, with a decrease in SUVmax of 0.02 per day.^70^

### Photoacoustic and optical coherence tomography (OCT) imaging

Photoacoustic imaging was evaluated in a phantom model using rat IVC thrombus. This imaging technique relies on the absorption of electromagnetic radiation and the emission of a thermoelastic acoustic wave, with red blood cells (RBCs) being the primary absorbers in DVT. The results showed that the magnitude of the radiofrequency signal was higher in acute DVT compared to mature DVT due to the increased RBC content in the acute thrombus.^71^

OCT operates on a similar principle to ultrasound but uses light instead of sound waves to generate images. In an in vivo rat IVC thrombus examination, birefringence showed 91.1% accuracy and 82.9% sensitivity for detecting acute thrombi, with intensity being significantly lower in chronic thrombi. A combined linear discriminant analysis achieved 98.2% accuracy, 97.6% sensitivity, and 98.6% specificity in distinguishing between thrombus types.^72^

## Discussion

The detection and classification of DVT chronicity remains a critical issue in vascular medicine. A wide range of imaging techniques including ultrasound, elastography, CT, MRI, nuclear medicine, photoacoustic imaging and OCT have been explored for this purpose. Over the years the number of studies attempting to use imaging to discriminate thrombus chronicity has increased significantly. A previous systematic review in 2015 identified only 15 relevant studies. This review identified 56 studies. In the previous review, MRI was not as widely studied compared to this review.^73^

Duplex ultrasound continues to be the most utilised modality for the detection of DVT. Consequently, it would be logical to investigate ultrasonographic features of DVT that could aid in determining thrombus chronicity, thereby potentially reducing the need for unnecessary supplementary investigations. However, as reported by this review, while certain features such as vein diameter and thrombus echogenicity may provide indications of thrombus age, they are ultimately inaccurate and unreliable indicators of thrombus chronicity. Furthermore, ultrasound is highly operator-dependent, and its effectiveness can be compromised in certain situations, such as when visualizing thrombi in challenging locations or in patients with obesity or significant swelling, which can limit its utility. The incorporation of elastography into ultrasound enhances the ability to assess thrombus age significantly. This review indicates that while elastography shows promising results, the limited number of studies and variability in the definitions of acute, subacute, and chronic thrombus pose challenges. Additionally, its availability is not universal, and its effectiveness is dependent on the operator. Nevertheless, existing evidence supports its capacity to detect changes that align with histological findings and to predict the success of CDT.

Among the imaging modalities reviewed, MRI emerges as the most promising for assessing thrombus chronicity, with some studies suggesting a sensitivity of 93% and specificity of 90%.^61^ MRDTI has gained increasing use in the routine evaluation of DVT in some centres, helping to guide treatment decisions, including the identification of patients who may benefit from CDT.^74^ However, due to the diversity of MRI techniques available, selecting the appropriate method for chronicity assessment requires careful consideration. Additionally, MRI interpretation requires specialised radiologists, and the technique is expensive, time-consuming, and less well-tolerated by certain patients. Furthermore, MRI availability may be limited in certain settings. Given these limitations, if MRI is to be used for chronicity assessment, it may need to be reserved for a select group of patients and its cost-effectiveness should be assessed.

Accurately determining thrombus age would permit identification of patients who would benefit from more interventional therapies, such as CDT and mechanical thrombectomy, potentially reducing the incidence of PTS. Additionally, it could help identify thrombi that may not require anticoagulation, thereby reducing the risk of anticoagulation-associated bleeding. In the long term, this approach could prevent complications such as ulceration, mitigate the socioeconomic burden associated with these conditions, and improve overall quality of life for patients.

Determining thrombus age is crucial; however, current imaging techniques, apart from MRI, may offer limited utility. This raises the question of whether there is a simple, relatively non-invasive method to accurately assess the chronicity of a DVT. Exploring novel modalities assessing thrombus biology in greater detail, such as high-throughput metabolomics or proteomics through translational platforms, could provide valuable insights.^75^ These approaches may help to not only diagnose and characterize thrombi but also offer prognostic information regarding their nature, potentially advancing the understanding of thrombus chronicity and improving patient outcomes.^76^

## Conclusion

This review analysed and evaluated the available evidence and utility of various thrombus aging imaging techniques. Whilst no single imaging technique appears to be ideal in determining the age of a thrombus, MRI provides greatest scope for advancement. If used in a select population, it may offer a cost-effective method to determine thrombus age and potentially improve the outcomes of catheter-based interventions for DVT.

## Supplemental Material


Supplemental Material - A systematic review evaluating imaging techniques to determine chronicity of deep vein thrombosis
Supplemental Material for A systematic review evaluating imaging techniques to determine chronicity of deep vein thrombosis by Kirtan D. Patel, Manal Ahmad, Matthew Tan, Sarah Onida, and Alun Davies in Phlebology.
