# Supplementary material for: A systematic review evaluating imaging techniques to determine chronicity of deep vein thrombosis
Source: Phlebology. 2025 Jul 16;41(3):179–89. doi: 10.1177/02683555251358915 (PMC12982563; doi:10.1177/02683555251358915)
Supplement: Supplemental Material - A systematic review evaluating imaging techniques to determine chronicity of deep vein thrombosis [file sj-pdf-1-phl-10.1177_02683555251358915.pdf]

Medline (via PubMed)

Search Group	Search Number	Search
DVT	1	(venous thrombosis[MeSH Terms]) OR (deep venous thrombosis[MeSH Terms])
	2	((DVT)[All Fields] OR (deep vein thrombos*s)[All Fields]) OR (deep venous thrombos?s)[All Fields]
	3	1 OR 2
Imaging	4	diagnostic imaging[MeSH Terms]
	5	Imaging[All Fields]
Ultrasound	6	(((ultrasonography[MeSH Terms]) OR (doppler ultrasonography[MeSH Terms])) OR (doppler ultrasonography, duplex[MeSH Terms])) OR (elasticity imaging techniques[MeSH Terms])
	7	(((Ultraso*)[All Fields] OR (Elastography)[All Fields]) OR (Doppler)[All Fields]) OR (Duplex)[All FIelds]
	8	6 OR 7
CT	9	tomography, x ray computed[MeSH Terms]
	10	(("CT")[All Fields] OR ("CTV")[All Fields]) OR ("CT venography")[All Fields]
	11	9 OR 10
MRI	12	magnetic resonance imaging[MeSH Terms]
	13	(((MRI)[All Fields] OR (MRV)[All Fields]) OR ("Magnetic resonance venography")[All Fields]) OR ("MR venography")[All fields]
	14	12 OR 13
Nuclear	15	radionuclide imaging[MeSH Terms]
Venography	16	phlebography[MeSH Terms]
	17	Venography[All Fields]
	18	16 OR 17
IVUS	19	((interventional ultrasonography[MeSH Terms]) OR (interventional ultrasound[MeSH Terms])) OR (intravascular ultrasonography[MeSH Terms])
	20	Intravascular Ultraso*[All Fields]
	21	19 AND 20
Aging	22	(((ag*ing)[All Fields] OR (staging)[All Fields]) OR (chronicity)[All Fields]) OR (acuity)[All Fields]
	23	4 OR 5 OR 8 OR 11 OR 14 OR 15 OR 18 OR 21
	24	3 AND 21 AND 23

Embase (OVID)

Search Group	Search Number	Search
DVT	1	exp vein thrombosis/ or exp deep vein thrombosis/
Imaging	2	exp diagnostic imaging/ or imaging.mp.
Ultrasound	3	exp ultrasound/
	4	exp echography/ or exp compression ultrasonography/ or exp contrast-enhanced ultrasound/ or exp doppler ultrasonography/ or exp elastography/ or exp high frequency ultrasound/
	5	exp duplex doppler ultrasonography/
	6	ultraso*.mp. [mp=title, abstract, heading word, drug trade name, original title, device manufacturer, drug manufacturer, device trade name, keyword heading word, floating subheading word, candidate term word]
	7	3 or 4 or 5 or 6
CT	8	exp computer assisted tomography/ or exp x-ray computed tomography/ or exp computed tomographic venography/
	9	("CT" or "CTV").mp. [mp=title, abstract, heading word, drug trade name, original title, device manufacturer, drug manufacturer, device trade name, keyword heading word, floating subheading word, candidate term word]
	10	8 or 9
MRI	11	exp nuclear magnetic resonance imaging/ or exp magnetic resonance elastography/ or exp magnetic resonance venography/
	12	("MRI" or "MRV").mp. [mp=title, abstract, heading word, drug trade name, original title, device manufacturer, drug manufacturer, device trade name, keyword heading word, floating subheading word, candidate term word]
	13	11 or 12
Nuclear	14	exp scintiscanning/
Venography	15	exp phlebography/ or exp leg phlebography/
	16	venography.mp. [mp=title, abstract, heading word, drug trade name, original title, device manufacturer, drug manufacturer, device trade name, keyword heading word, floating subheading word, candidate term word]
	17	15 or 16
IVUS	18	exp interventional ultrasonography/
	19	exp intravascular ultrasound/
	20	IVUS.mp. [mp=title, abstract, heading word, drug trade name, original title, device manufacturer, drug manufacturer, device trade name, keyword heading word, floating subheading word, candidate term word]
	21	18 or 19 or 20
Imaging	22	2 or 7 or 10 or 13 or 17 or 21
Aging	23	(ag?ing or staging or acuity or chronicity).mp. [mp=title, abstract, heading word, drug trade name, original title, device manufacturer, drug manufacturer, device trade name, keyword heading word, floating subheading word, candidate term word]
	24	1 and 22 and 23

Web of Science

Search Number	Search
1	ALL=(DVT OR "deep vein thrombos?s" OR "deep venous thrombos?s" OR "venous thrombos?s")
2	ALL=(diagnostic imaging OR imaging)
3	ALL=(Ultraso* OR Doppler OR Duplex OR Elasticity OR Elastography)
4	ALL=(CT OR CTV OR CT venogra* OR computeri?ed tomography)
5	ALL=(MRI or MRV or MR venography or magnetic resonance imaging or magnetic resonance venography)
6	ALL=(Radionuclide imaging OR Nuclear imaging)
7	ALL=(phlebography OR Venography)
8	ALL=(interventional ultraso* or intravascular ultraso* or IVUS)
9	#2 OR #3 OR #4 OR #5 OR #6 OR #7 OR #8
10	ALL=(ag?ing OR Staging or acuity or chronicity)
11	#1 AND #9 AND #11

CENTRAL (Cochrane)

Search Group	Search Number	Search
DVT	1	MeSH descriptor: [Venous Thrombosis] explode all trees
	2	(DVT):ti,ab,kw
	3	(deep vein thrombos?s OR deep venous thrombos?s):ti,ab,kw
	4	#1 OR #2 OR #3
Imaging	5	MeSH descriptor: [Diagnostic Imaging] explode all trees
	6	(imaging):ti,ab,kw
	7	#5 OR #6
Ultrasound	8	MeSH descriptor: [Ultrasonography] explode all trees
	9	MeSH descriptor: [Ultrasonography, Doppler] explode all trees
	10	MeSH descriptor: [Ultrasonography, Doppler, Duplex] explode all trees
	11	MeSH descriptor: [Elasticity Imaging Techniques] explode all trees
	12	(Elastography OR Ultraso* OR Doppler OR Duplex):ti,ab,kw
	13	#8 OR #9 OR #10 OR #11 OR #12
CT	14	MeSH descriptor: [Tomography, X-Ray Computed] explode all trees
	15	(CT OR CTV OR CT venogra*):ti,ab,kw
	16	#14 OR #15
MRI	17	MeSH descriptor: [Magnetic Resonance Imaging] explode all trees
	18	(MRI OR MRV OR MR venogra* OR Magnetic resonance venogra*):ti,ab,kw
	19	#17 OR #18
Nuclear	20	MeSH descriptor: [Radionuclide Imaging] explode all trees
Venography	21	MeSH descriptor: [Phlebography] explode all trees
	22	(venography):ti,ab,kw
	23	#21 OR #22
IVUS	24	MeSH descriptor: [Ultrasonography, Interventional] explode all trees
	25	(Intravascular Ultraso* OR Interventional Ultraso* OR IVUS):ti,ab,kw
	26	#24 OR #25
Imaging	27	#7 OR #13 OR #16 OR #19 OR #20 OR #23 OR #26
Aging	28	(ag?ing OR staging OR acuity OR chronicity):ti,ab,kw
	29	#4 AND #27 AND #28
